# Geographic Elevation and Cognitive Function among Elderly Residents in Rural Mountainous Areas: Shimane CoHRE Study

**DOI:** 10.3390/ijerph121013365

**Published:** 2015-10-23

**Authors:** Tsuyoshi Hamano, Keiichi Onoda, Miwako Takeda, Kristina Sundquist, Shuhei Yamaguchi, Toru Nabika

**Affiliations:** 1 Center for Community-Based Health Research and Education (CoHRE), Organization for the Promotion of Project Research, Shimane University, 223-8 Enya-cho, Izumo, Shimane 693-8501, Japan; E-Mails: cohre1@med.shimane-u.ac.jp (M.T.); nabika@med.shimane-u.ac.jp (T.N.); 2 Department of Neurology, Shimane University School of Medicine, 89-1 Enya-cho, Izumo, Shimane 693-8501, Japan; E-Mails: onodak1@med.shimane-u.ac.jp (K.O.); yamagu3n@med.shimane-u.ac.jp (S.Y.); 3 Center for Primary Health Care Research, Lund University, Clinical Research Centre (CRC), Building 28, floor 11, Jan Waldenströms gata 35, Skåne University Hospital, SE-205 02 Malmö, Sweden; E-Mail: kristina.sundquist@med.lu.se; 4 Stanford Prevention Research Center, Stanford University, Medical School Office Building (MSOB), 251 Campus Drive MC 5411, Stanford, CA 94305, USA; 5 Department of Functional Pathology, Shimane University School of Medicine, 89-1 Enya-cho, Izumo, Shimane 693-8501, Japan

**Keywords:** cognitive function, elevation, rural mountainous area, elderly people

## Abstract

The aim of this study was to test whether there is an association between elevation and cognitive function among elderly residents in rural mountainous areas. Data were collected in 2012 from a cross-sectional study conducted in Ohnan Town, which is located in a rural mountainous area in the southern part of Shimane Prefecture, Japan. Cognitive function was evaluated using CADi (Cognitive Assessment for Dementia, iPad version) and elevation was estimated by using Geographic Information Systems according to the participant’s address. After excluding subjects with missing data, 866 participants were analyzed. After adjustment for potential confounding factors, higher elevation was significantly associated with decreased cognitive function. This finding suggests that it is important to consider the physical environment, *i.e.*, elevation, that would affect accessibility to health-promoting goods, services, and resources when seeking to maintain cognitive function in elderly people living in rural mountainous areas.

## 1. Introduction

Decreasing cognitive function is a major public health concern as the population ages [1]. Several risk factors for cognitive function have been postulated, including sociodemographic factors (e.g., age and education), lifestyle factors (e.g., smoking and diet), and health conditions (e.g., current history of a disease) [2,3,4,5,6,7,8,9]. More recently, research on the association between the physical environment, e.g., geographic elevation, and age-related diseases has gained momentum, with an increased focus on Geographic Information Systems (GIS) [10,11,12,13,14,15]. 

A previous study conducted in Japan found that people living at higher elevations were more likely to have decreased masticatory ability [12]. Additionally, people living at higher elevations had a higher salt intake than those living at lower elevations [10]. In Japan, hilly and mountainous areas occupy approximately 70% of the country and are defined as regions extending from the outer plains to the mountains [16]. Health-promoting goods, services, and resources, which may have a positive effect on cognitive function, are substantially limited at higher elevations as compared to lower elevations. It is therefore hypothesized that elderly people who live at higher elevations have lower cognitive function compared to those who live at lower elevations. To the best of our knowledge, no previous study has investigated the hypothesized associations. 

The aim of this study was to test whether there is an association between elevation and cognitive function among elderly residents in rural mountainous areas. Cognitive function was evaluated using CADi (Cognitive Assessment for Dementia, iPad version), which can be run on a tablet computer for mass screening in the community [17].

## 2. Methods

### 2.1. Study Design

Data were collected from a cross-sectional study conducted in 2012. The present study was a part of the Shimane CoHRE Study, which was designed to examine the determinants of age-related diseases, including cognitive function [10,11,12,13,14,15,17]. The Shimane CoHRE study was conducted by Shimane University, Japan, in collaboration with a health examination program that involved Ohnan Town. This town is located in a rural mountainous area in the southern part of Shimane Prefecture, Japan. 

Health examination programs are available once a year for individuals between 40 and 74 years of age who are covered by National Health Insurance. The residents in this town have two options when they wish to undergo a health examination: They may participate in a group examination conducted at public health centers, or they may receive an individual examination conducted at medical institutions. We were permitted to use data on group examinations for our analysis. After excluding participants with missing data (36 participants), we analyzed data from 866 participants. The study protocol was approved by the Ethics Committee of Shimane University School of Medicine in 2010. Written informed consent was obtained from all participants.

### 2.2. Cognitive Function

Cognitive function was assessed using CADi which has been evaluated on validity and reliability [17]. CADi consists of 10 separate items: immediate recognition of three words, question about semantic memory, categorization of six objects, subtraction, backward repetition of three digits, cube rotation, pyramid rotation, trail-making test A, trail-making test B, and delayed recognition of three words. The score ranges from 0 to 10, with higher scores indicating better cognitive function. The participants answered during the voice presentation of a question statement using an iPad (https://itunes.apple.com/us/app/cadi/id586052447, currently Japanese version only).

### 2.3. Elevation

Geographic Information Systems (ArcGIS software, version 10.0, Environmental Systems Research Institute, Redlands, CA, USA) was employed for database queries and used to estimate elevation based on the individuals’ addresses. The elevation for each participant was assessed using the ArcGIS ready-to-use dataset of digital elevation models (mean value = 252.8 m; range, 72 m to 465 m). 

### 2.4. Other Measures

We considered the following variables in the analysis: age (years, analyzed as a continuous variable), gender (male *vs.* female), body mass index (BMI) (kg/m^2^, analyzed as a continuous variable), current smoker (yes *vs*. no), current alcohol drinker (yes *vs*. no), physically active (engaged in physical activity regularly = yes *vs*. not engaged in physical activity regularly = no), use of medication for disease treatment (medication to reduce blood pressure, medication to reduce blood sugar or insulin injections, and medication to reduce cholesterol level), accessible transportation (driver = yes *vs*. non-driver = no), and a self-rating depression scale (SDS) (analyzed as a continuous variable).

### 2.5. Statistical Analysis

Descriptive statistics were calculated. Multivariable linear regression models were performed to derive regression coefficients (B), standard errors (SE), and *p*-values. Independent variables were coded as follows: Gender (0 = male, 1 = female), and current smoker, current alcohol drinker, regular physical activity, medication for disease treatment, and accessible transportation (0 = no, 1 = yes). *P*-values less than 0.05 were considered statistically significant. All statistical analyses were performed using IBM SPSS Statistics 20 (IBM Corporation, Tokyo, Japan).

## 3. Results

The characteristics of the study participants are shown in Table 1. The study consisted of 866 participants (41.2% male and 58.8% female) and the mean (standard deviation: SD) age was 65.8 (7.7) years. The mean (SD) CADi score was 8.1 (1.5). Of the participants, 110 (12.7%) were smokers, and 461 (53.2%) reported drinking alcohol. Around 30% of participants were physically active; 303 participants (35.0%) took medication for hypertension and 664 participants (76.7%) were drivers. The mean (SD) BMI and SDS (SD) were 22.7 (3.1) kg/m^2^ and 35.0 (8.2), respectively. 

**Table 1:** Characteristics of the study participants.

Variables	Number of Participants	% or Mean (SD)
CADi score, mean (SD)	866	8.1 (1.5)
Elevation, m (SD)	866	252.8 (74.5)
Age, years (SD)	866	65.8 (7.7)
Gender (male), %	357	41.2
Current smoker, %	110	12.7
Current alcohol drinker, %	461	53.2
Regular physical activity, %	280	32.3
Medication		
To reduce blood pressure, %	303	35.0
To reduce blood sugar or insulin injections, %	55	6.4
To reduce cholesterol level, %	191	22.1
Body Mass Index, kg/m^2^	866	22.7 (3.1)
Driver, %	664	76.7
SDS, mean (SD)	866	35.0 (8.2)

CADi, Cognitive Assessment for Dementia, iPad version; SD, standard deviation; SDS, self-rating depression scale.

Table 2 shows the results of the multivariable liner regression analysis. Higher elevation was significantly associated with decreased cognitive function (regression coefficient = −0.002, *p* = 0.016). Age, gender, medication for hypertension, and being a driver were also significantly associated with cognitive function (regression coefficient = −0.044, *p* < 0.001; regression coefficient = 0.345, *p* = 0.004; regression coefficient = −0.313, *p* = 0.007; regression coefficient = 0.718, *p* < 0.001, respectively).

## 4. Discussion

To the best of our knowledge, no study has examined the association between elevation and cognitive function among elderly people. As we hypothesized, our results showed that higher elevation is significantly associated with decreased cognitive function. These results suggest that elevation could be an important determinant of cognitive function in rural mountainous areas. 

**Table 2:** Multivariable linear regression analysis with cognitive function as dependent variable.

Variables	B	SE	*t*	*p*-Value
Age	−0.044	0.007	−6.367	<0.001
Gender	0.345	0.119	2.894	0.004
Current smoker	0.220	0.162	1.360	0.174
Current alcohol drinker	0.037	0.109	0.335	0.738
Regular physical activity	−0.055	0.108	−0.506	0.613
Medication				
To reduce blood pressure	−0.313	0.115	−2.711	0.007
To reduce blood sugar or insulin injections	0.028	0.210	0.132	0.895
To reduce cholesterol level	0.211	0.129	1.633	0.103
Body Mass Index	−0.028	0.017	−1.721	0.086
Driver	0.718	0.126	5.693	<0.001
SDS	−0.008	0.006	−1.324	0.186
Elevation	−0.002	0.001	−2.419	0.016

SDS, self-rating depression scale.

This study advances the recent debate on the association between the physical environment, *i.e.*, elevation, and health. As noted, a previous study found that people living at higher elevations are more likely to have decreased masticatory ability [12]. Also, overweight individuals living at a high elevation had a higher risk of chronic knee pain than non-overweight individuals living at a low elevation [13]. The mechanisms underlying the association between elevation and health outcomes are most likely of a complex nature, and causal inferences remain to be established. Considering that our participants live in a rural mountainous area, people living at higher elevations may be more exposed to the effects of relatively limited access to health-promoting goods, services, and resources than those living at lower elevations. For example, previous research conducted in rural mountainous areas found that people living at higher elevations had a higher salt intake than those living at lower elevations [10]. Further studies are required to examine whether health behaviors differ by the elevation in rural mountainous areas.

Decreasing cognitive function is a major public health concern as the population ages [1]. Health professionals should manage efficient health plans that can select appropriate target groups for interventions. Our results suggest that people living at higher elevations should be considered a priority target to measure cognitive function. CADi, which was used in this study, is a useful instrument for mass screening in the community due to its low cost, brief administration time, and user-friendliness for elderly people [17], so understanding the physical environment, *i.e.*, elevation, and using CADi can help health professionals promote efficient activities in the community.

There are a number of potential limitations in the current study. First, our data set from group examination may not be an entirely representative sample. A selection bias caused by non-respondents was present and may have influenced the associations shown in our results. Caution is therefore warranted in over-interpreting this finding. However, the participation rate of the group examination (37.1%) was higher than that of the individual examination (19.1%) and 43.8% of residents did not receive health examinations during this year. Second, misclassification may have occurred in the self-reported data as a consequence of recall errors. However, we have no reason to believe that this potential bias differed between the residential areas. Third, our data did not allow for the assessment of other important confounding factors such as education and income. Finally, the present study used a cross-sectional design that did not allow us to establish temporal ordering or causal relationships.

## 5. Conclusions 

Our analysis indicated that high elevation was significantly associated with decreased cognitive function. This finding suggests that it is important to consider the physical environment, *i.e.*, elevation, when seeking to maintain cognitive function in the elderly living in rural mountainous areas because this may affect their accessibility to health-promoting goods, services, and resources. Longitudinal research is needed to confirm these findings and explore the underlying mechanisms.
